# Reasons for Physicians’ Tendency to Irrational Prescription of Corticosteroids

**DOI:** 10.5812/ircmj.2284

**Published:** 2012-11-15

**Authors:** Nazila Yousefi, Reza Majdzadeh, Mahboube Valadkhani, Saharnaz Nedjat, Hanie Mohammadi

**Affiliations:** 1 School of Public Health, Tehran University of Medical Science, Tehran, Iran; 2 Iran Food & Drug Organization, Deputy of Ministry of Health and Medical Education, Tehran, Iran; 3 Knowledge Utilization Research Center (KURC), Tehran University of Medical Science, Tehran, Iran

**Keywords:** Inappropriate Prescriptions, Corticosteroids, Qualitative Research

## Abstract

**Background:**

According to World Health Organization (WHO) estimation, more than half of all pharmaceutical products are inappropriately prescribed, distributed, and sold and more than half of all patients use the medicines prescribed for them incorrectly. As more than 40% of therapeutic costs are pharmaceutical costs, this implies a significant waste of health resources in the world.

**Objectives:**

To find effective factors in irrational prescription of corticosteroids in Iran and design suitable interventions to decrease prescription rates of corticosteroids.

**Materials and Methods:**

A qualitative study was performed in 2009 on fifteen general practitioners in two groups identified by high and low corticosteroid prescription rates. Data analysis was performed by thematic analysis and the study's validity was based on training interviewers, use of interview guide, avoidance of imposing opinions, coding by two independent persons and use of all opinions obtained in the analysis.

**Results:**

The effective factors in irrational prescription of corticosteroids can be divided into four categories: lack of knowledge, patient-physician relationship in terms of monetary cost, poor availability of proper alternative medicines and weak supervision of regulatory bodies. As the same results were found in both groups regarding the role of regulatory organizations and availability of alternative medicines, it seems that interventions in knowledge and the patient-physician relationship which were different in the two groups can be more effective for reduction of prescription in high rate prescribers although intervention in regulatory supervision and medicine availability could have a moderate effect in both groups. In addition the common feature in all the above categories was the gap between knowledge and actual practice which is significant on three regulatory levels, supervisors, physicians and patients, and should be noted for intervention design.

**Conclusions:**

The interventions applied in other countries can also be effective in decreasing irrational prescription of corticosteroids in Iran. These interventions include: standard clinical guidelines, essential medicines list, practical workshops, purposeful training based on problem-solving, training of all parties including pharmacists and patients, improved regulatory mechanisms, availability of assured quality medicines, availability of suitable alternatives to painkillers and realistic rational prescription policy.

## 1. Background

According to World Health Organization (WHO) estimation, more than half of all pharmaceutical products are inappropriately prescribed, distributed, and sold and more than half of all patients use the medicines prescribed for them incorrectly. As more than 40% of therapeutic costs are pharmaceutical costs, this implies a significant waste of health resources in the world. In addition, according to WHO estimation, each year, between 2.3 and 4.7 million new cases of hepatitis and 160000 cases of AIDS occur owing to 15 billion injections of which more than 90% are unnecessary. Inappropriate drug prescription leads to delay in correct diagnosis and treatment, drug resistance, increase in drug interactions and adverse events. According to WHO's 2001 definition, rational drug use means that patients receive medications appropriate to their clinical needs, in doses that meet their own requirements, for an adequate period of time and at the lowest cost to them and their community (1). Since 1985 when WHO held a large conference regarding rational drug use in Nairobi (2), many efforts have been made globally to improve the status of rational prescription of medicines.

In Iran, prescription data are collected continuously by the Prescription Control Center. According to this center’s report, Dexamethasone injection was the most prescribed medication from 2006 to 2009. On the basis of the fourth IRAN socio-economic developmental program (for the years 2005 to 2009), it was planned that corticosteroids prescription would be reduced from 20% to 16% and a further 6% reduction was planned for the next program (3), which clearly was not achieved because corticosteroid prescription in Iran was 24% for 2008, an increase of 6% compared with three years earlier (4). Also according to the Iranian Ministry of Health, more than 5 billion pain killers including corticosteroids, NSAID and acetaminophen were used in Iran in 2007 (5) which was 1.8 times more than Denmark, 3.3 times more than Norway and 1.7 times more than UK (6).

Since corticosteroids, which are generally known as Cortisone, alleviate the signs and symptoms of disease quickly, despite having long- and short-term side-effects such as hypertension, weight gain, decreased immunity and increased risk of infections, osteoporosis, mood swings, increase in blood sugar, cataract, insomnia, delay in wound healing, depression and hallucination, edema, cardio-vascular diseases, and delay the diagnosis and treatment of diseases, they are quite popular with doctors and patients (7). Corticosteroids directly add to undesired costs in the health system by increasing its administration and indirectly by prolonging the duration of treatment and causing side-effects, a significant factor. In a quantitative study performed in Iran in 2006, the reasons for irrational use of injection medicines were into account but other medicine groups such as corticosteroids have not been studied (8).

## 2. Objectives

This study was designed and performed to investigate the effective factors leading to irrational and high rates of corticosteroid prescription by physicians.

## 3. Materials and Methods

A qualitative study was performed in 2009 (April to October) on two groups of general practitioners. Interviewees were chosen by extreme case sampling from two groups of general practitioners which according to the Prescription Control Center’s data in 2008 had the highest and lowest rates of corticosteroid prescription respectively Lists of general practitioners, number of prescriptions and percentage of prescribed corticosteroids were extracted. The list includes physicians in both private and governmental sectors. Physicians were listed according to the rate of corticosteroid prescription, ranging from high to low. The average rate of prescription by these physicians was 23%, with the lowest being 0.3% and the highest being 81%. The contact number and address of physicians in both groups was acquired from the Medical Council and ten physicians from both groups who agreed to be interviewed, whose offices were located in Tehran and who did not specialize in certain fields that could affect prescription rate of corticosteroids were chosen. In qualitative research, sample size is not predefined and would be determined based on data saturation during the study. In current study following fifteen interviews (seven in the high rate group and eight in the low rate group), data saturation was reached, interviews were discontinued and the information was organized. Data collection by semi-structured interviews was performed by two interviewers (one pharmacist and one physician) who were trained in interview skills and informed about the goals of this study, and each interview took about one hour. Interviews were noted, recorded and fully described on paper at the end of each. During the interviews, the imposition of any preconceived ideas was meticulously avoided. The interviewers had sufficient basic knowledge of corticosteroid indications and contra-indications and the objectives of the study. Interviews were performed according to a pre-designed interview guide includes some open questions based on the study objectives. Interviews were performed by appointment at the physician’s office outside peak working hours. Furthermore, a closed questionnaire was completed with regard to the common sources used to obtain information, work experience and graduation date, number of patients per day and the estimated amount of prescriptions by the physicians themselves.

Qualitative analysis was done by the thematic analysis method. Data coding was done by two members of the research team and themes were extracted. By means of open coding, a code was dedicated to each sentence. In case of disagreement, decisions were made by a third party. Descriptions were studied, key sentences were extracted, results were aggregated in clusters and themes were extracted. The viewpoint of both groups was compared and the reliability of the study was based on training the interviewers, using an interview guide, avoiding imposition of certain ideas, coding by two independent persons and use of all opinions obtained in the analysis. As regards ethical issues, at the time of each interview, after a general description of the objectives of the study, permission was asked to record the physicians' voices and it was explained that they could stop the recording at any time they wished. Personal information was not used in stating the results and the names were replaced by codes in the analysis tables. The proposal of this study was approved by the School of Public Health of the Tehran University of Medical Sciences. The proposal review process is under the supervision of the Committee of Ethics in Research, Tehran University of Medical Sciences (which follows the principles of the Declaration of Helsinki).

## 4. Results

The range of prescription was 2 to 13% (average 7%) in the group with low rates and 49 to 61% (average 53%) in the high rate group. Personal and professional characteristics of the two groups did not differ significantly for items such as average age (43 and 42), average work experience (15 and 12 years), annual training hours (about 25) and number of patients per day (40).

The four main themes extracted were as follows:

1- Poor physician’s knowledge of correct treatment

2- Patient- physician relationship

3- Unavailability of suitable alternative medicines

4- Improper supervision by regulatory bodies.

The opinions expressed in both study groups and some of the key sentences which had a more prominent role in theme extraction are mentioned below.

Poor Physician’s Knowledge Of Correct Treatment High prescription group: the rate of studying reference books was low and the rate of incorrect pharmaceutical information was high. Also there is no proper scientific understanding regarding indications and method of administration of corticosteroids. Physicians observed that: “General practitioners have a lot of problems in the job and do not have any time to study,” “80% of treatments mentioned in Harrison’s text book involve cortisones,” “Cortisone is effective on all patients,” “I do not remember a certain dose,” “Based on experience, I prescribe one fourth of an ampoule for children.”

Although this group was familiar with the side-effects of corticosteroids it did not pay much attention to them in practice: “I have never seen any side-effects.” This group believed that advertisements did not affect them: “Advertisements are effective on lay people.” They received most of their information, however, from pharmaceutical companies: “Reference textbooks are useful in university and are not applicable in practice. Promotional brochures and the internet are more beneficial.” Low prescription group: this group use reference textbooks more often. They even use textbooks to inform patients: “I spend more time and describe the complications to the patient from Farsi textbooks.” The physicians of this group believe that advertisements do not affect them: “company representatives only highlight the benefits and therefore are unreliable.”

### 4.1. Patient-Physician Relationship

High prescription group: in this group, competition to attract more patients for financial gain is more prominent: “There are many general practitioners and too much competition,” “Patients are keen to get well quickly and I do not want to lose my patient,” “If I do not prescribe this medicine, somebody else will,” “When a child’s fever ameliorates with one injection, his/her parents will be very satisfied.” The patient’s occupational problems also play a role: “A worker is eager to get well soon in order to go back to work,” “Injection cortisone is in demand in this region,” “I do not deny any patient’s request.”

Patients do not receive the necessary pharmaceutical information and in some cases even receive falsely optimistic information. “If the patient becomes aware of the complications, he will discontinue therapy on his own accord,” “Some people are afraid of cortisones but I explain that nothing will happen to them.” In addition, patient knowledge affects medicine prescription: “If the patient does not receive injection cortisones, she thinks nothing has been done for her.” Low prescription group: they think there is competition to attract patients for financial gain, so symptom therapy has replaced correct treatment because it is easier and patients are more satisfied with fast alleviation of symptoms. They believe in correct treatment: “A patient who is not correctly treated will not come back to the same physician again.”

Therefore, safe and healthy competition should replace unhealthy competition: “Proper scientific practice is the best advertisement.” On the other hand, the prevailing culture in society should be improved: “People like injection medicines,” “If I do not prescribe it, somebody else will or the patient will obtain it from the pharmacy.” If the patient trusts his physician, however, he will not insist on his request: “The patient’s request is not important to me; I follow the correct treatment regimen and try to obtain the patient’s confidence.”

### 4.2. Unavailability of Suitable Alternative Medicines

The major classes of analgesics are parastamol, NSAIDs, COX2 inhibitors, Opiates and morphinomimetics, Flupirtine and some specific agents in other groups of medicines. There are several combinations and formulations of above mentioned groups to introduce effective medicines. NSAIDs and corticosteroids are most available groups in Iran.

High prescription group: lack of suitable alternative painkillers in Iran, especially in terms of appropriate injection forms and the variable quality of the present dosage forms on the market, was mentioned as the main reason for corticosteroid prescription: “The local generic oral painkillers are ineffective and brand products are expensive,” “Even topical dosage forms are ineffective and we prescribe injection corticosteroids for topical disorders,” “If a suitable injection painkiller becomes available on the market, use of corticosteroids will decrease.”

Low prescription group: in this group, lack of suitable alternative painkillers in Iran, especially in terms of appropriate injection forms and the variable quality of the present pharmaceutical products on the market, is also mentioned as a primary reason for corticosteroid prescription: “There is no other substitute and the physician is obliged to use corticosteroids,” “Non-steroid painkillers available on the market are of poor quality and ineffective,” “Migraine headaches and other severe pains cannot be treated with prayer so injection corticosteroids should be administered.”

### 4.3. Improper Supervision by Regulatory Bodies

High prescription group: they believe that the patients’ culture and response to therapy are different in our country, and therefore national therapeutic guidelines which are confirmed by related regulatory organizations are needed. There is no appropriate supervision of the sale of medicines without prescription and they can be obtained easily from pharmacies: “Pharmacists are only keen on selling medicines and are not held responsible, general practitioners always are.”

There is no active supervision system for medicine prescription: “There is only strict control over insurance prescriptions for financial reasons,” “There is no notification system in place for physicians from the Prescription Control Center.”

Low prescription group: this group also sees the necessity for therapeutic guidelines from related organizations. They also believe that a large part of medicine administration is because of sales without prescription by pharmacies which are not properly controlled: “Inspectoral organizations are all talk and no action.” There is no active inspectoral system for rational prescription of medicines. Physicians are not aware of the criteria for rational use of medicines and “there is no persuasive/punitive mechanism or any training for this purpose.” Factors affecting medicine prescription are complex and a combination of socio-cultural beliefs influenced by knowledge, background and economic factors. For effective intervention, initially these factors should be identified and analyzed (9).

These factors should be taken into consideration on three levels: macro (legislators and programmers), meso (management and supervision), and micro (physicians and patients) (10). In the present study, factors at macro level include: unclear policies and lack of rational prescription criteria, lack of national therapeutic guidelines and protocols, inappropriate supervision of all sections of the therapeutic cycle such as pharmacists and pharmaceutical companies, lack of practical educational programs, lack of a suitable medicine quota, lack of high-quality oral painkillers which leads to inappropriate prescription of corticosteroids. At meso level: lack of active supervision of medicine prescription and failure to apply persuasive/punitive methods, financial issues of general practitioners, and promotional activities of pharmaceutical companies. At micro level: physician’s knowledge, patient-physician relationship, physician’s ability to apply his/her knowledge properly, the influence of colleagues and other physicians' behavior. Furthermore, factors such as the medicine distribution chain, medicine prices, quality of medicines (even their physical appearance and packaging) (11), national drug prescription regulations and many other factors such as the Iranian Drug List, availability of medicines and pricing methods (12) may influence drug prescription patterns.

As rational prescription is influenced by cultural, social and economic factors which may be different in each society, qualitative study is more convenient for discovering the underlying reasons; in similar studies performed quantitatively, for example, certain factors reviewed in this study were missed. For this purpose, investigational methods such as exploratory and theory-inspired theories have been proposed. The advantage of theory-free methods is that they avoid being limited by theories and can pay attention to unpredictable issues. The advantage of theory-based methods is that there is no possibility of missing important issues. The best option is to use both methods simultaneously to discover and design interventions (13). Both methods have been used in the present study. Initially the exploratory or theory-free method was used and responses were received and categorized without the use of a particular template. The acquired results can be adapted with theories which in turn will be beneficial in designing interventions in later stages.

### 4.4. Knowledge

A systematic review study in 2007 showed that one of the main reasons for inappropriate drug prescription is the lack of reliable information resources or lack of availability of these data for physicians, nurses and patients (14). In the USA, pharmaceutical companies with annual sales of 216 billion dollars, spending more than 21 billion dollars on advertisement and marketing, play the most important role in drug information notification (15). Most of these advertisements lead to unnecessary, expensive, addictive over-administration of medicines. In the present study, the difference in the level of knowledge and information resources between the two studied groups highlights this effect on the use of medicines. The low prescription group made more use of reference textbooks in their medical practice, but the high prescription group had incorrect pharmaceutical information and mostly acquired information from pharmaceutical companies, unlike the other group. Although both groups strongly denied the effect of advertisement, according to the results of this study and similar articles mentioned, the influence of information resources and advertisements of pharmaceutical companies on inappropriate administration of pharmaceutical products is undeniable. In a study performed in India, the most important reason for irrational use of medicines was found to be the marketing activities of pharmaceutical companies and lack of strict regulations by the Ministry of Health (16). Results from a study in the USA showed that the reasons for over-prescription of painkillers were patients’ desire for a quick remedy and physicians’ tendency towards an easier therapeutic approach rather than spending time on training and behavioral change (17). In the present study too, lack of patients’ knowledge of their therapeutic advantages and disadvantages and patients' and physicians' tendency towards easy and fast recovery are some of the reasons for irrational prescription of corticosteroids.

### 4.5. Effect of Patients and Other Medical Groups

Studies demonstrate that besides beliefs regarding therapeutic methods in terms of efficacy and adverse events, the physician’s belief about colleagues’ behavior is effective on therapeutic behavior. In the light of this, in order to put scientific knowledge into practice, we have to start with physicians; intervention should start with physicians having more influence on their own network (18). One of the themes considered in this study is the nature of relations between the physician and patient and other medical groups. Although physicians’ financial insecurity and competition to attract patients exist in both groups, they are more prominent in the high prescription group. Anxiety about losing patients is higher in the high prescription group, as they believe that if they do not prescribe a fast-acting injection painkiller, other physicians or pharmacists will and they will lose the patient. According to this study's results, however, the number of patients in the two groups is equal, because the low prescription group believes in attracting patients by presenting accurate information and correct treatment instead of using a fast therapeutic approach and meeting patients’ medicine requests.

### 4.6. Availability of Suitable Drug Alternatives

Availability of a wide range of high-quality pharmaceutical products is one of the requirements for rational prescription. In the present study both groups believe that suitable painkillers are unavailable on the market and oral or topical painkillers do not have proper efficacy and quality. Both groups are eager to have suitable injection painkillers on the market. Although the aim is to reduce the administration of painkillers, especially injection forms, and only the introduction of a new injection painkiller will change the administration pattern, the variable quality of available non-injection painkillers and use of injection painkillers in all instances are worth taking into account.

### 4.7. Role of Regulatory and Supervisory Organizations

In a study performed in a number of low- and middle-income countries, the reasons for physicians’ irrational drug prescription were as follows: lack of written and effective guidelines, lack of effective inspection and supervision and lack of clear policy (19). In the performed studies, education, drug policies and practical guidelines and therapeutic protocols were estimated to be effective in improving the status of rational prescription (20). In the present study, both groups criticized the incompetent inspectoral system with regard to unclear policies and criteria for rational prescription, poor compilation and notification of national therapeutic guidelines, lack of active supervision of drug prescription and use of persuasive/punitive methods. Furthermore, they asked for proper inspection of all parts of the therapeutic system including pharmacists and drug companies.

### 4.8. Knowledge and Practice Gap

The gap between scientific knowledge and practice is the common element among the four themes and should be considered on all levels - patients, physicians and legislators. The high prescription group thought that there was no abuse of corticosteroids. Although they stated that “a maximum 10% of patients referring to general practitioners require corticosteroids,” their average prescription was 54%. The low prescription group, who also believed in correct practice methods, observed that “with the current economic and occupational conditions, you cannot expect general practitioners to practice scientifically.”

In addition, the data collected for the question “how much do you prescribe corticosteroids?” were for the higher group 23% (standard deviation=26) less than the actual rate and for the lower group 0.6% (standard deviation=7) less than the actual rate. The gap between scientific knowledge and practice is not only limited to physicians but also exists at all levels from patients to legislators. For instance, in a study performed in Canada, of the eight policy procedures studied, only one of them was designed according to the studies' results (13).

Interventions including choosing behavioral change technology and moving from current behavior to science-oriented behavior following identification of effective agents should be. These interventions should be assessable and consist of at least three essential factors, plausibility, feasibility and efficacy, (21) and four steps: applying change, evaluation of procedure, monitoring the scale of effect in the short and long term. Although these solutions are particularly appropriate for individuals who are aware of the problem and willing to obviate it, in all cases they can be effective with a little modification.

## 5. Discussion

Rational prescription and use of medicines are influenced by different factors such as lifestyles and habits, the culture of medicine use, the knowledge and culture of healthcare personnel and regulatory organizations' supervision and policies. Factors effective in inappropriate prescription of corticosteroids can be divided into four general categories: scientific knowledge, patient-physician relationship, availability of suitable medicine alternatives and function of inspectoral organizations. According to these results, the interventions used in other countries can also be effective in decreasing the irrational prescription of corticosteroids in Iran. These interventions include: standard clinical guidelines, essential drugs list, practical workshops, purposeful training based on problem-solving, training of all parties, including pharmacists and patients, improved regulatory mechanisms, availability of assured quality medicines, availability of suitable painkiller alternatives and realistic rational prescription policy. As the same results were found in both groups regarding the role of regulatory organizations and availability of alternative medicines, it seems that interventions in knowledge and patient-physician relationships, which were different in the two groups, can be more effective for reduction of prescription in high-rate prescribers although the intervention in regulatory supervision and medicine availability could have a moderate effect in both groups. In addition the common feature in all the above categories was the gap between knowledge and actual practice which is significant on three regulatory levels, supervisors, physicians and patients, and should be noted in intervention design.
